# Supplementary material for: Risk factors for gallstone disease onset in Japan: Findings from the Shizuoka Study, a population-based cohort study
Source: PLoS One. 2022 Dec 30;17(12):e0274659. doi: 10.1371/journal.pone.0274659 (PMC9803237; doi:10.1371/journal.pone.0274659)
Supplement: S1 Table — (DOCX) [file pone.0274659.s001.docx]

**S1 Table.** Insurance claim codes for gallstone-related medical interventions

| **Medical intervention** | **Japanese claim code** |
| --- | --- |
| Gallbladder incision lithotomy | 150172210 |
| Choledocholithotomy (including cholecystectomy) | 150296610 |
| Choledocholithotomy (excluding Laparoscopic cholecystectomy) | 150172310 |
| Laparoscopic choledocholithotomy (including cholecystectomy) | 150276810 |
| Laparoscopic choledocholithotomy (excluding cholecystectomy) | 150276910 |
| Laparoscopic cholecystectomy | 150254110 |
| External cholecystostomy | 150174110 |
| External biliary drainage (Surgery) | 150174210 |
| External biliary drainage (percutaneous transhepatic) | 150174310 |
| Percutaneous bile duct drainage | 150174550 |
| Endoscopic naso-biliary drainage | 150362310 |
| EUS-guided biliary drainage | 150362410 |
| Endoscopic stone extraction technique | 150174910 |
| Percutaneous transhepatic gallbladder aspiration | 150341350 |
| Endoscopic sphincterotomy | 150175410 |
| Endoscopic papillary balloon dilation：EPBD | 150341450 |
| Endoscopic biliary stent placement | 150254410 |
| Percutaneous transhepatic bile duct stent placement | 150263810 |
| Percutaneous transhepatic biliary baloon dilation | 150401310 |
