# Supplementary material for: Risk factors for gallstone disease onset in Japan: Findings from the Shizuoka Study, a population-based cohort study
Source: PLoS One. 2022 Dec 30;17(12):e0274659. doi: 10.1371/journal.pone.0274659 (PMC9803237; doi:10.1371/journal.pone.0274659)
Supplement: S2 Table — ALT, alanine aminotransferase; AST, aspartate aminotransferase; BMI, body mass index; GFR, glomerular filtration rate; GGT, gamma-glutamyl transpeptidase; HbA1c, hemoglobin A1c; HDL, high-density lipoprotein; LDL, low-density lipoprotein. (DOCX) [file pone.0274659.s002.docx]

**S2 Table.** Characteristics of individuals with gallstones who underwent treatment for the disease and those who did not undergo treatment

| **Variable** | **Category or unit** |  |  | **P value** |
| --- | --- | --- | --- | --- |
|  |  | **No treatment** | **Treatment** |  |
|  |  | N= 19084 | N= 4759 |  |
| Sex | Women | 10666 (55.9) | 2131 (44.8) | <0.001 |
|  | Men | 8418 (44.1) | 2628 (55.2) |  |
| Age | 0 to <40 years | 33 (0.2) | 8 (0.2) | <0.001 |
|  | 40 to <50 years | 523 (2.7) | 97 (2.0) |  |
|  | 50 to <60 years | 1015 (5.3) | 241 (5.1) |  |
|  | 60 to <70 years | 6364 (33.3) | 1415 (29.7) |  |
|  | 70 to <80 years | 7060 (37.0) | 1920 (40.3) |  |
|  | 80 years to | 4089 (21.4) | 1078 (22.7) |  |
| **Comorbidities** |  |  |  |  |
| Cerebrovascular disease | Presence | 3423 (17.9) | 839 (17.6) | 0.636 |
| Any malignancy | Presence | 1913 (10.0) | 473 (9.9) | 0.882 |
| Dementia | Presence | 599 (3.1) | 154 (3.2) | 0.767 |
| Myocardial infarction | Presence | 351 (1.8) | 106 (2.2) | 0.091 |
| Renal disease | Presence | 536 (2.8) | 124 (2.6) | 0.475 |
| Rheumatic disease | Presence | 611 (3.2) | 110 (2.3) | 0.002 |
| Liver disease | Presence | 41 (0.2) | 6 (0.1) | 0.293 |
| Congestive heart failure | Presence | 2187 (11.5) | 521 (10.9) | 0.332 |
| Chronic pulmonary disease | Presence | 4287 (22.5) | 971 (20.4) | 0.002 |
| Diabetes | Presence | 1210 (6.3) | 345 (7.2) | 0.025 |
| Hypertension | Presence | 10602 (55.6) | 2823 (59.3) | <0.001 |
| Pylori-infected gastritis | Presence | 1237 (6.5) | 276 (5.8) | 0.09 |
| History of gastrectomy | Presence | 16 (0.1) | 2 (0.0) | 0.519 |
| HIV infection | Presence | 1 (0.0) | 0 (0.0) | 1 |
| Crohn’s disease | Presence | 14 (0.1) | 2 (0.0) | 0.664 |
| Spinal injury | Presence | 1 (0.0) | 0 (0.0) | 1 |
| Total parenteral nutrition | Presence | 0 (0.0) | 0 (0.0) | NA |
| **Medical checkup** |  |  |  |  |
| Frequency of alcohol consumption | Never | 9847 (63.1) | 2331 (59.7) | <0.001 |
|  | Occasionally | 2838 (18.2) | 727 (18.6) |  |
|  | Everyday | 2916 (18.7) | 845 (21.7) |  |
| Use of lipid-lowering agents | Yes | 5469 (28.7) | 1349 (28.4) | 0.684 |
| Current smoker | Yes | 1893 (9.9) | 499 (10.5) | 0.256 |
| Walking or physical exercise > 1 h / wk | Yes | 8499 (49.8) | 2011 (48.5) | 0.138 |
| Increase in weight > 10 kg since age of 20 y | Yes | 5617 (32.9) | 1731 (41.6) | <0.001 |
| Estimated GFR | mL/min/1.73 m^2^ | 66.58 (16.07) | 65.76 (15.90) | 0.002 |
| BMI | kg/m^2^ | 22.98 (3.54) | 23.82 (3.51) | <0.001 |
| GGT | U/L | 38.01 (59.35) | 42.90 (89.42) | <0.001 |
| AST | U/L | 25.63 (13.57) | 25.76 (14.71) | 0.562 |
| ALT | U/L | 21.41 (15.76) | 22.31 (19.33) | 0.001 |
| HbA1c | % | 5.78 (0.70) | 5.87 (0.82) | <0.001 |
| LDL cholesterol | mg/dL | 120.86 (30.87) | 120.34 (31.05) | 0.294 |
| Systolic blood pressure | mmHg | 130.42 (16.53) | 131.83 (16.75) | <0.001 |
| Triglycerides | mg/dL | 117.17 (70.38) | 124.35 (71.78) | <0.001 |
| Uric acid | mg/dL | 5.31 (1.34) | 5.50 (1.33) | <0.001 |

ALT, alanine aminotransferase; AST, aspartate aminotransferase; BMI, body mass index; GFR, glomerular filtration rate; GGT, gamma-glutamyl transpeptidase; HbA1c, hemoglobin A1c; HDL, high-density lipoprotein; LDL, low-density lipoprotein.
