# Supplementary material for: Risk factors for gallstone disease onset in Japan: Findings from the Shizuoka Study, a population-based cohort study
Source: PLoS One. 2022 Dec 30;17(12):e0274659. doi: 10.1371/journal.pone.0274659 (PMC9803237; doi:10.1371/journal.pone.0274659)
Supplement: S3 Table — ALT, Alanine aminotransferase; AST, aspartate aminotransferase; BMI, body mass index; GFR, glomerular filtration rate; GGT, gamma-glutamyl transpeptidase; HbA1c, hemoglobin A1c; LDL, low-density lipoprotein. (DOCX) [file pone.0274659.s003.docx]

**S3 Table.** Spearman’s correlation matrix

|  | **Age** | **Cerebro-**  **vascular**  **disease** | **Any**  **malignancy** | **Dementia** | **Myocardial**  **infarction** | **Renal**  **disease** | **Rheumatic**  **disease** | **Liver**  **disease** | **Congestive**  **heart**  **failure** | **Chronic**  **pulmonary**  **disease** | **Frequency**  **and**  **volume**  **per day**  **of drink** | **Sex** | **Use of**  **lipid-**  **lowering**  **agents** | **Current**  **smoker** |
| --- | --- | --- | --- | --- | --- | --- | --- | --- | --- | --- | --- | --- | --- | --- |
| **Cerebrovascular disease** | 0.240 | 1 |  |  |  |  |  |  |  |  |  |  |  |  |
| **Any malignancy** | 0.120 | 0.043 | 1 |  |  |  |  |  |  |  |  |  |  |  |
| **Dementia** | 0.192 | 0.136 | 0.013 | 1 |  |  |  |  |  |  |  |  |  |  |
| **Myocardial infarction** | 0.069 | 0.071 | 0.022 | 0.016 | 1 |  |  |  |  |  |  |  |  |  |
| **Renal disease** | 0.095 | 0.063 | 0.043 | 0.039 | 0.050 | 1 |  |  |  |  |  |  |  |  |
| **Rheumatic disease** | 0.046 | 0.022 | 0.015 | 0.009 | 0.008 | 0.022 | 1 |  |  |  |  |  |  |  |
| **Liver disease** | 0.015 | 0.011 | 0.031 | 0.009 | 0.003 | 0.015 | 0.005 | 1 |  |  |  |  |  |  |
| **Congestive heart failure** | 0.216 | 0.155 | 0.069 | 0.086 | 0.209 | 0.141 | 0.042 | 0.018 | 1 |  |  |  |  |  |
| **Chronic pulmonary disease** | 0.095 | 0.064 | 0.046 | 0.023 | 0.022 | 0.034 | 0.044 | 0.006 | 0.095 | 1 |  |  |  |  |
| **Frequency and volume of**  **alcohol consumption**  **per day** | 0.133 | 0.046 | 0.001 | 0.063 | 0.016 | 0.016 | 0.039 | 0.007 | 0.039 | 0.032 | 1 |  |  |  |
| **Sex** | -0.005 | -0.031 | -0.075 | 0.019 | -0.069 | -0.050 | 0.049 | -0.009 | -0.041 | 0.007 | 0.412 | 1 |  |  |
| **Use of lipid-lowering agents** | -0.157 | -0.141 | -0.008 | -0.004 | -0.092 | -0.039 | -0.019 | 0.008 | -0.088 | -0.054 | -0.109 | -0.104 | 1 |  |
| **Current smoker** | -0.192 | -0.059 | -0.040 | -0.036 | -0.016 | -0.018 | -0.023 | -0.001 | -0.046 | -0.026 | -0.185 | -0.235 | 0.084 | 1 |
| **Walking or physical exercise**  **> 1 h / wk** | -0.063 | 0.017 | -0.005 | 0.058 | 0.004 | 0.014 | 0.013 | 0.002 | 0.031 | 0.023 | 0.035 | 0.029 | 0.012 | 0.033 |
| **Increment in weight**  **> 10 kg since age of 20 y** | 0.098 | -0.010 | 0.010 | 0.029 | -0.016 | -0.002 | 0.015 | 0.000 | -0.012 | -0.026 | 0.053 | 0.127 | 0.081 | -0.046 |
| **Estimated GFR** | -0.416 | -0.140 | -0.065 | -0.087 | -0.070 | -0.172 | -0.014 | -0.007 | -0.162 | -0.044 | -0.088 | 0.039 | 0.128 | 0.126 |
| **BMI** | -0.046 | 0.027 | -0.013 | -0.039 | 0.025 | 0.011 | -0.017 | -0.001 | 0.032 | 0.019 | -0.065 | -0.168 | -0.127 | 0.015 |
| **GGT** | -0.118 | -0.007 | 0.006 | -0.055 | 0.017 | -0.004 | -0.015 | 0.016 | 0.005 | 0.006 | -0.358 | -0.380 | -0.018 | 0.170 |
| **AST** | 0.122 | 0.016 | 0.036 | -0.009 | 0.010 | -0.007 | 0.018 | 0.024 | 0.028 | 0.026 | -0.107 | -0.095 | -0.08 | -0.036 |
| **ALT** | -0.161 | -0.029 | -0.007 | -0.073 | 0.003 | -0.037 | -0.01 | 0.012 | -0.036 | -0.005 | -0.123 | -0.219 | -0.085 | 0.049 |
| **HbA1c** | 0.141 | 0.061 | 0.033 | 0.006 | 0.054 | 0.020 | 0.005 | 0.000 | 0.057 | 0.028 | 0.086 | -0.038 | -0.200 | -0.030 |
| **LDL cholesterol** | -0.136 | -0.113 | -0.050 | -0.032 | -0.087 | -0.057 | -0.02 | -0.025 | -0.119 | -0.048 | 0.099 | 0.123 | 0.216 | -0.032 |
| **Systolic blood pressure** | 0.248 | 0.047 | 0.010 | 0.017 | 0.001 | 0.014 | 0.001 | 0.002 | 0.027 | -0.007 | -0.044 | -0.049 | -0.058 | -0.039 |
| **Triglycerides** | 0.008 | 0.009 | -0.005 | -0.004 | 0.015 | 0.021 | -0.005 | -0.006 | 0.010 | 0.002 | -0.029 | -0.127 | -0.095 | 0.107 |
| **Uric acid** | 0.039 | 0.038 | 0.026 | -0.012 | 0.044 | 0.077 | -0.017 | 0.007 | 0.076 | 0.013 | -0.249 | -0.451 | 0.010 | 0.113 |
| **Diabetes** | 0.081 | 0.086 | 0.050 | 0.014 | 0.076 | 0.063 | 0.014 | 0.015 | 0.101 | 0.025 | 0.018 | -0.063 | -0.102 | -0.012 |
| **Hypertension** | 0.360 | 0.243 | 0.061 | 0.075 | 0.103 | 0.109 | 0.034 | 0.014 | 0.225 | 0.099 | 0.000 | -0.049 | -0.259 | -0.074 |
| **Pylori infected gastritis** | 0.017 | 0.016 | 0.046 | -0.012 | 0.001 | 0.009 | 0.009 | 0.014 | 0.005 | 0.021 | -0.009 | -0.017 | -0.022 | -0.013 |
| **History of gastrectomy** | 0.007 | 0.002 | 0.066 | -0.001 | 0.002 | 0.006 | 0.001 | 0.002 | 0.009 | 0.000 | 0.002 | -0.010 | 0.002 | -0.002 |

**S3 Table.** Spearman’s correlation matrix (continued)

|  | **Current**  **smoker** | **Walking**  **or physical**  **exercise**  **> 1 h / wk** | **Increment**  **in weight**  **> 10 kg**  **since the**  **age of 20 y** | **Estimated**  **GFR** | **BMI** | **GGT** | **AST** | **ALT** | **HbA1c** | **LDL**  **cholesterol** | **Systolic**  **blood**  **pressure** | **Triglycerides** | **Uric**  **acid** | **Diabetes** | **Hypertension** | **Pylori-**  **infected**  **gastritis** |
| --- | --- | --- | --- | --- | --- | --- | --- | --- | --- | --- | --- | --- | --- | --- | --- | --- |
| **Cerebrovascular disease** |  |  |  |  |  |  |  |  |  |  |  |  |  |  |  |  |
| **Any malignancy** |  |  |  |  |  |  |  |  |  |  |  |  |  |  |  |  |
| **Dementia** |  |  |  |  |  |  |  |  |  |  |  |  |  |  |  |  |
| **Myocardial infarction** |  |  |  |  |  |  |  |  |  |  |  |  |  |  |  |  |
| **Renal disease** |  |  |  |  |  |  |  |  |  |  |  |  |  |  |  |  |
| **Rheumatic disease** |  |  |  |  |  |  |  |  |  |  |  |  |  |  |  |  |
| **Liver disease** |  |  |  |  |  |  |  |  |  |  |  |  |  |  |  |  |
| **Congestive heart failure** |  |  |  |  |  |  |  |  |  |  |  |  |  |  |  |  |
| **Chronic pulmonary disease** |  |  |  |  |  |  |  |  |  |  |  |  |  |  |  |  |
| **Frequency and volume of**  **alcohol consumption**  **per day** |  |  |  |  |  |  |  |  |  |  |  |  |  |  |  |  |
| **Sex** |  |  |  |  |  |  |  |  |  |  |  |  |  |  |  |  |
| **Use of lipid-lowering agents** |  |  |  |  |  |  |  |  |  |  |  |  |  |  |  |  |
| **Current smoker** | 1 |  |  |  |  |  |  |  |  |  |  |  |  |  |  |  |
| **Walking or physical exercise**  **> 1 h / wk** | 0.033 | 1 |  |  |  |  |  |  |  |  |  |  |  |  |  |  |
| **Increment in weight**  **> 10 kg since age of 20 y** | -0.046 | -0.066 | 1 |  |  |  |  |  |  |  |  |  |  |  |  |  |
| **Estimated GFR** | 0.126 | 0.016 | 0.018 | 1 |  |  |  |  |  |  |  |  |  |  |  |  |
| **BMI** | 0.015 | 0.043 | -0.583 | -0.076 | 1 |  |  |  |  |  |  |  |  |  |  |  |
| **GGT** | 0.170 | 0.019 | -0.230 | 0.045 | 0.261 | 1 |  |  |  |  |  |  |  |  |  |  |
| **AST** | -0.036 | -0.042 | -0.053 | -0.062 | 0.056 | 0.354 | 1 |  |  |  |  |  |  |  |  |  |
| **ALT** | 0.049 | -0.011 | -0.244 | 0.074 | 0.292 | 0.516 | 0.662 | 1 |  |  |  |  |  |  |  |  |
| **HbA1c** | -0.030 | -0.005 | -0.154 | -0.063 | 0.204 | 0.109 | 0.049 | 0.147 | 1 |  |  |  |  |  |  |  |
| **LDL cholesterol** | -0.032 | 0.010 | -0.051 | 0.030 | 0.072 | 0.001 | -0.048 | 0.029 | 0.033 | 1 |  |  |  |  |  |  |
| **Systolic blood pressure** | -0.039 | -0.015 | -0.093 | -0.089 | 0.182 | 0.107 | 0.109 | 0.063 | 0.092 | 0.024 | 1 |  |  |  |  |  |
| **Triglycerides** | 0.107 | 0.057 | -0.250 | -0.101 | 0.335 | 0.276 | 0.058 | 0.209 | 0.168 | 0.189 | 0.129 | 1 |  |  |  |  |
| **Uric acid** | 0.113 | 0.006 | -0.203 | -0.308 | 0.279 | 0.332 | 0.141 | 0.210 | 0.082 | -0.009 | 0.101 | 0.248 | 1 |  |  |  |
| **Diabetes** | -0.012 | 0.000 | -0.039 | -0.057 | 0.058 | 0.016 | -0.002 | 0.027 | 0.265 | -0.089 | 0.032 | 0.023 | 0.028 | 1 |  |  |
| **Hypertension** | -0.074 | 0.002 | -0.111 | -0.239 | 0.188 | 0.076 | 0.082 | 0.029 | 0.151 | -0.179 | 0.270 | 0.103 | 0.142 | 0.122 | 1 |  |
| **Pylori infected gastritis** | -0.013 | -0.008 | 0.003 | -0.011 | -0.001 | 0.012 | 0.022 | 0.018 | 0.006 | -0.006 | -0.007 | -0.006 | 0.003 | 0.013 | 0.021 | 1 |
| **History of gastrectomy** | -0.002 | -0.001 | 0.005 | 0.001 | -0.012 | 0.001 | 0.009 | 0.008 | 0.002 | -0.016 | -0.002 | -0.008 | 0.000 | 0.011 | 0.007 | 0.014 |

ALT, alanine aminotransferase; AST, aspartate aminotransferase; BMI, body mass index; GFR, glomerular filtration rate; GGT, gamma-glutamyl transpeptidase; HbA1c, hemoglobin A1c; LDL, low-density lipoprotein.
